# Xenotransplantation could either be a friend or foe of healthcare equity

**DOI:** 10.1038/s43856-024-00511-0

**Published:** 2024-05-11

**Authors:** Marie Chisholm-Burns, Burnett S. Kelly, Christina A. Spivey

**Affiliations:** 1 Oregon Health & Science University, 3225 S.W. Pavilion Loop, MC: L101 Baird Hall (Suite 1011), Portland, OR 97239 USA; 2 DCI Donor Services Inc., 3940 Industrial Blvd, West Sacramento, CA 95691 USA

**Keywords:** Health care, Organ transplantation

## Abstract

Chisholm-Burns et al. discuss the substantial shortage of organs available for transplantation, with disparities in access amongst some racial and ethnic groups. The authors suggest that while xenotransplantation can potentially increase organ availability, it also has the potential to further embed inequities in transplant care.

## Teaser

There are critical shortages in donor organs available for transplantation in the United States, with some racial and ethnic groups facing disparities in access. Xenotransplantation is currently an experimental approach, but has the promise of significantly increasing organ supply. Here, we discuss inequities in access to transplantation and the potential role of xenotransplantation in alleviating these inequities.

## Introduction

In the United States, there is a critical shortage of donor organs available for transplant. As of April 2024, there were over 103,800 individuals on the U.S. waitlist for transplant, compared to 46,630 transplants completed in 2023^1^. The allocation of donor organs for transplantation is therefore a balancing act of the principles of justice (defined as “fair consideration of candidates’ circumstances and medical needs”) and medical utility (defined as “trying to increase the number of transplants performed and the length of time patients and organs survive”)^2,3^. More specifically, Bunnik^3^ points to an ethical struggle in organ allocation due to competing interests such as severity of illness, expected benefit of transplant (i.e. posttransplant prognosis), and time on waitlist. In addition to these three factors, other considerations in the process of allocating organs include donor-recipient blood type matching, immune system matching, and organ size matching, as well as geographic location^2^. A further challenge to the organ allocation system is the documented disparities in access to solid-organ transplantation faced by some racial and ethnic groups including Black, Hispanic, Asian/Pacific Islander, and American Indian and Alaska Native patients^4^. Xenotransplantation, the transplant of non-human animal organs into humans, may 1 day play a role in addressing shortages in donor organs and resolving challenges of organ allocation. Due to anatomical and physiological similarities to humans (including organ size), domestic pigs are considered the most appropriate donors for xenotransplantation^5^. Pigs used for xenotransplantation are raised in laboratory settings and genetically modified to reduce the risk of organ rejection following transplant into humans^5^. However, despite recent clinical advances, as mentioned below, the science of xenotransplantation is still in its infancy.

This Comment discusses equitable access in solid organ transplantation and the potential role of xenotransplantation, and considers the following: the opportunity presented by xenotransplantation in helping to achieve equitable, high-quality transplant care for racial and ethnic minority groups, as well as the risk that utilization of xenotransplantation will further propagate structural biases that could result in a stratified system where patients belonging to racial and ethnic minority groups are more likely to be disadvantaged than non-Hispanic white patients. In this way, xenotransplantation is emblematic of the global health equity issues we face when attempting to innovate and then allocate resource-limited healthcare treatment options to disparate populations.

## Transplant care inequities

When considering the path of a patient with end-stage organ disease to a potential treatment, there are several barriers that impair equitable access to transplant healthcare^4^. Two such barriers, implicit bias and structural racism, may be major contributors to solid-organ transplant care disparities. For example, as noted by Tsai et al.^6^, one structural impediment to transplant referral was the use of poorly substantiated glomerular filtration rate (GFR) calculators that overestimated GFR in Black patients (due to documented disparities, the Organ Procurement & Transplantation Network finally implemented a requirement for race-neutral estimated GFR calculations in July 2022)^7^. In turn, disparities in care may lead to worse overall morbidity and mortality from treatable end-stage organ disease^4^.

In the U.S.A., Black, Hispanic/Latinx, and other minority patients are less likely to be referred for transplant evaluation^4,8^ When referred, these patients are less likely to be waitlisted for transplant^4,8^. If waitlisted, Black and Hispanic/Latinx patients are more likely to be listed as “inactive” (a patient may be listed as inactive, or ineligible to receive a transplant, for a variety of factors including medical illness or psychosocial issues, among others), have greater waitlist mortality or health decline, and greater time on the waitlist, which may ultimately result in poorer post-transplant health outcomes – if a patient is fortunate enough to receive a transplant^4,8–11^. Racial and ethnic minority patients, particularly Black patients, are also less likely to receive both deceased and living donor organ transplants, thus having fewer opportunities for the standard of care for transplant organ options^4,8^. Improvements in health equity across the continuum of transplantation are clearly needed.

## The role of xenotransplantation and challenges in its equitable adoption

Xenotransplantation aspires to be the next medical frontier, offering therapeutic solutions to patients with end-stage organ disease. Yet despite progress in genetic engineering of more immune-compatible pigs as donors, the question remains how to best operationalize xenotransplantation from experimental to mainstream practice to address current organ access disparities and inherent inequities in the organ allocation system, without compromising patient outcomes^12^. In an ideal scenario, the introduction of a supply of pig organs that could be produced just-in-time for transplant and function as well as current standards of human organ donation would alleviate pressures on the allocation system (and may potentially eliminate the need for an allocation system). It would also shift the public health priority to organ manufacturing, distribution, identifying suitable patients with end-stage organ disease, and developing a workforce with the capabilities of caring for xenotransplant patients.

However, the science of xenotransplantation remains in its early stages. In 2022, the first pig-to-human heart transplant was performed in a 57 year-old man with end-stage heart disease who was denied a human heart transplant based on concerns he was not a suitable candidate^13^. He did not leave the hospital postoperatively and died of complications within 2 months of the xenotransplant. Although he was fully consented for the potential risks and many would describe this first xenotransplant as an innovative success, his outcome was far below the expected survival from a human heart transplant. More recently, in March 2024, a 62 year-old man (whose previous human kidney transplant failed after 5 years) received the world’s first pig kidney transplant and was discharged 2 weeks later to continue his recovery from home; as of this writing, he remains in good condition^14^.

So, to continue advancing this innovation, who should be the research participants? Medical history would suggest that medical advances often occur through the efforts of vulnerable populations with limited treatment options^15^. There are thus several challenges in developing an equitable, high-quality xenotransplant system. The first challenge is determining the functional quality of transplanted pig organs as better or worse than human organs, or good enough to justify consideration as a viable treatment modality versus no transplant. The second challenge is surveying the public perception regarding the use of pig organs (such as hearts and kidneys) in humans, particularly in vulnerable populations with above-average waiting times and no other identified organ source to minimize excessive clinical deterioration on the waitlist. The third challenge is creating appropriate clinical trials to address proof of transplant benefit in vulnerable populations given the hesitancy, mistrust, and historically low participation of some racial and ethnic minority groups in clinical trials. It is during the initial proof of concept and practice adoption phases where these aggregate challenges could most significantly (and potentially deleteriously) impact the most vulnerable patients with the highest need for transplant: racial and ethnic minority patients. In the absence of effectively and transparently addressing these challenges and disseminating the results to potential patients, transplant professionals, ethicists, researchers, insurance payors, and regulatory agencies, it is unclear how xenotransplantation will not create a tiered system of transplant care where the most vulnerable patients will continue to go underserved. To explain further, a tiered system may result wherein the most vulnerable patients receive the most vulnerable donor organs, ie, the most accessible organs with a potentially poorer post-transplant prognosis as compared to the alternative organ option – whether they be through traditional transplant care or xenotransplantation. During this window of experimental uncertainty, the higher clinical urgency of racial and ethnic minority patients for transplant may dictate decision-making and limit access to the established standard of care. This selection bias could then consequently have the downstream effect of decreasing the willingness of racial and ethnic minority patients to accept either organ option for transplant, thus propagating the disparity.

## A path to equitable xenotransplantation

The challenges of organ allocation do not have simple solutions. As long as there is a persistent disparity between the number of people on the waitlist and number of available organs, equity issues will be of foremost concern in relation to who, when, and how one receives an organ^1^. Xenotransplantation is not a new technology, but the clinical application is still experimental^16^. Xenotransplantation has the promise of providing more organs at a quicker pace, theoretically significantly increasing access to solid-organ transplantation in racial and ethnic minority groups who historically have had longer waiting times and higher waitlist dropout rates.

Despite the aforementioned advances in genetic pig engineering, several serious ethical considerations have been identified and were the focus of a 2023 American Transplant Congress session entitled, “Ethical Concerns in Pig to Human Xenotransplantation^17–19^”. These ethical considerations include animal rights concerns, public health and infectious disease risks, and questions regarding justice and equity in organ access and use of resources^17–19^. Not specifically addressed during this session were the implications of xenotransplantation in relation to transplant inequities experienced by racial and ethnic minorities. It is not unreasonable to postulate that xenotransplantation could represent one strategy in addressing inequities in organ allocation. However, specifically targeting predominately racial and ethnic minorities for xenotransplantation at the potential risk of inequitable access to human donor transplant (the standard of care) would serve to create a new disparity in transplant care.

How then do we support xenotransplantation as a prospective innovative treatment option without creating more, new, or different inequities? As an initial step, protocols in xenotransplantation clinical trials should be evaluated to ensure adequate representation of all populations. Racial and ethnic minorities are typically underrepresented in clinical trials^20^. As a result, there are often knowledge gaps regarding safety and efficacy of new treatments in these populations. Inequitable representation in xenotransplantation clinical trials could serve as an initial disparity that becomes magnified when determining allocation policies for equitable pig or human organ access. Equitable representation in clinical trials would therefore help to advance safe and efficacious application of xenotransplantation as a treatment option for all patients. Furthermore, equitable representation would be essential to building trust in Black, Hispanic/Latinx, and other racial and ethnic populations that pig and human organ transplant outcomes will be equivalent. Dixon and Wilkins^21^ suggest comprehensive strategies to improve equitable representation in clinical trials including, but not limited to: training researchers to design equitable research protocols; establishing inclusive eligibility criteria; and creating targets for recruitment that reflect the racial and ethnic diversity of the patient population.

Following successful clinical trials, equitable, transparent, and patient-centered care algorithms of transplant benefit should be employed and consider the spectrum of the transplant process including referral, evaluation, allocation, and care. Healthcare providers should partner with patients throughout the process to build trust and ensure the patient understands risks and benefits. If the patient is not comfortable proceeding with xenotransplantation, healthcare providers should ensure they are appropriately referred to the standard care. This type of approach would assure patients are neither singled out for xenotransplantation nor denied equitable access to all viable transplant pathways.

Embracing xenotransplantation and building a better solid-organ transplantation process for tomorrow will also require bolstering the public’s trust in the current transplant process. We urge healthcare systems to address structural inequities in existing solid-organ transplantation processes and practices. The National Academies of Sciences, Engineering, and Medicine suggests a multi-modal approach to pursue equity in transplantation including: (a) creating and assessing measures of inequity to facilitate prompt action in redressing disparities; (b) adopting payment and reimbursement policies that create incentives to facilitate equity; (c) implementing more rigorous patient-centered care to build patient-provider trust and communication, and foster opportunities to address barriers in transplant care and improve outcomes; (d) increasing public education about the transplant system to strengthen public trust; and (e) uplifting the voices of those affected by disparities when developing transplant policies so that concerns about inequities can be better understood and eliminated^4^. Such strategies should be deployed in the current transplant milieu and later recalibrated to include xenotransplantation.

## Conclusions

The field of solid-organ transplantation must innovate to achieve optimal care, practices, and patient outcomes. However, progress should be made in alignment with principles of equity and justice and with an intentional approach to eliminate structural inequities and health disparities. Whether xenotransplantation ultimately becomes a friend or foe in promoting equity in transplantation remains to be seen. We must therefore be focused on pursuing equity in all corners of transplant care.
